# Preparation of Red TiO_2_ with Excellent Visible Light Absorption from Industrial TiOSO_4_ Solution for Photocatalytic Degradation of Dyes

**DOI:** 10.1021/acsomega.4c09262

**Published:** 2024-12-18

**Authors:** Hong Pu, Congxue Tian, Hui Zhang

**Affiliations:** † College of Chemistry and Chemical Engineering, Southwest Petroleum University, Chengdu 610500, China; ‡ Vanadium and TitaniumResource Comprehensive Utilization Key Laboratory of Sichuan Province, Panzhihua University, Panzhihua 617000, China

## Abstract

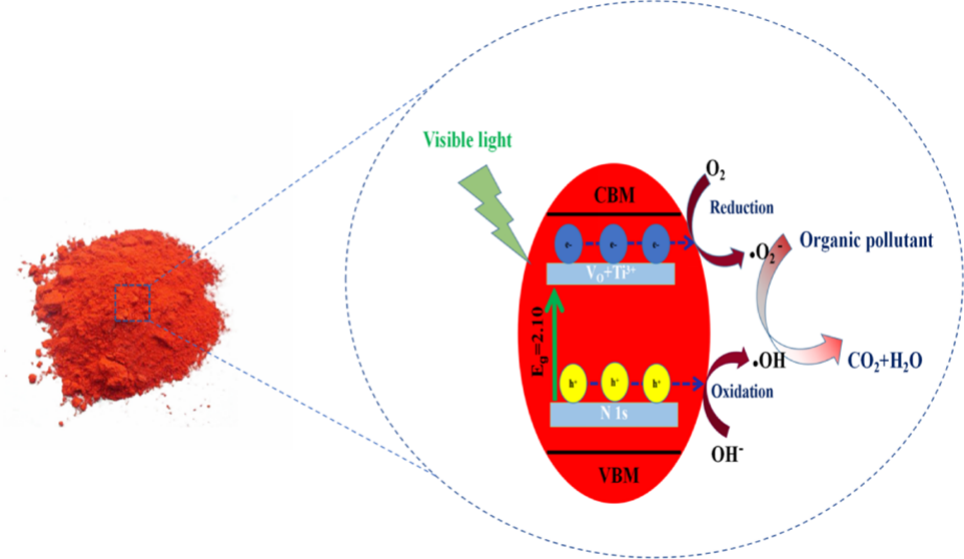

At present, it is still difficult to significantly reduce the bandgap of TiO_2_ to promote its visible light absorption. Herein, we first synthesized sulfur-doped TiO_2_ from industrial TiOSO_4_ and then successfully synthesized red TiO_2_ nanoparticles by calcination with the N source melamine. Theoretical calculations show that predoped S could markedly decrease the formation energy and substitution energy of N-doped TiO_2_, especially in high N/Ti ratios. The red TiO_2_ nanoparticles have a low bandgap (2.10 eV) and exhibit remarkable visible light absorption capacity. Electron paramagnetic resonance measurements show that the red TiO_2_ has abundant oxygen vacancies and Ti^3+^. The synergetic effect of Ti^3+^, oxygen vacancies, and nonmetallic element doping leads to the bandgap of TiO_2_ significantly being reduced. In addition, the red TiO_2_ exhibits great photocatalytic activity in the visible light degradation of rhodamine B (Rh.B) and methylene blue (MB). This study provides a new idea for the preparation of TiO_2_ with high visible light absorption.

## 1 Introduction

In the last few decades, TiO_2_ has been considered to be one of the most promising catalysts due to its nontoxicity, strong oxidation properties, and cost-effectiveness.^1−5^ Unfortunately, the photocatalytic capacity of TiO_2_ is very limited on account of its broad bandgap, which can only exhibit catalytic activity under ultraviolet light.^6,7^ Nonmetallic element (e.g., C, N, S, F, and P) doping is an effective way to improve the photocatalytic activity of TiO_2_.^8−12^ These nonmetal elements can reduce the bandgap of TiO_2_ through forming impurity energy levels and improving its visible light absorption. Meanwhile, defects such as Ti^3+^ and oxygen vacancies can also improve the photocatalytic activity of TiO_2_ effectively.^13,14^ Oxygen vacancies can introduce an electron state vacancy band below the conduction band, thereby reducing the band gap,^15^ while Ti^3+^ can promote local excitation by achieving a three-dimensional transition from the gap state to the empty excited state inside the material.^16^

Asahi et al.^17^ reported that N-doping could dramatically increase the light absorption and activity of TiO_2_. Since then, the visible light catalytic activity of nitrogen-doped TiO_2_ has been widely explored.^18−22^ Nitrogen ions have a similar size to oxygen ions (0.171 nm for N^3–^ ions and 0.132 nm for O^2–^ ions) and a small ionization energy.^23^ Nitrogen doped into TiO_2_ can form an interstitial nitrogen or substitutional nitrogen. Substituted N always causes the 2p levels of N and O to mix, thus narrowing the bandgap of TiO_2_. However, interstitial N may produce a middle state higher than the maximum VB, which could also reduce the bandgap.^24^

Experimental and theoretical calculations demonstrate that the nitrogen concentration significantly affects the optical properties of TiO_2_.^25−27^ Lower nitrogen doping levels result in only a slight narrowing of the bandgap. However, there is an obvious bandgap narrowing at high N doping concentrations, especially with homogeneous doping in bulk TiO_2_, where the dopant and the TiO_2_ have full and long-range coupling and could reduce the band gap effectively.^28^ So far, the synthesis of high N-doping in bulk TiO_2_ remains challenging in experiments due to the substantially higher formation energy needed for the process. Surprisingly, Liu et al. gave a new insight by predoping interstitial boron into TiO_2_ and then doping with nitrogen, red TiO_2_ was synthesized, which exhibited full visible light spectrum absorbance.^29^ The red color of TiO_2_ is due to deep layer nitrogen doping and oxygen vacancy. Is there any other element predoping that can also promote the doping of nitrogen into TiO_2_? Research shows that sulfur could be doped into the TiO_2_ lattice and change its geometric structure.^30^ Usually, the substitution of the Ti^4+^ ions (ionic radius, 0.068 nm) with the S^6+^ ions (ionic radius, 0.029 nm) is chemically more favorable compared to the substitution of the O^2–^ ions (ionic radius, 0.132 nm) with the S^2–^ ions (ionic radius, 0.17 nm). Yang et al. used first-principles calculations to show that sulfur easily replaces Ti^4+^ ions in the TiO_2_ lattice.^31^

In this work, the S-doped TiO_2_ nanoparticles were synthesized hydrothermally from an industrial TiOSO_4_ solution, and then, nitrogen doping was realized by using a simple high-temperature calcination process. The prepared nanoparticles (particle size of about 7 nm) showed a red color and had excellent visible light absorption. First-principles simulations showed that the presence of sulfur in TiO_2_ can significantly reduce the formation energy of nitrogen doping. This may be because the presence of sulfur in TiO_2_ can weaken the bonding energy of Ti–O bonds and contribute to nitrogen doping. The electron paramagnetic resonance test shows that red TiO_2_ has high oxygen vacancy and Ti^3+^. The excellent visible light absorption of red TiO_2_ results from the synergetic effect of oxygen vacancies, Ti^3+^, and nitrogen doping. This study is advantageous for the preparation of TiO_2_ with enhanced visible light absorption capabilities.

## 2 Experimental Section

### 2.1 Chemicals and Materials

The industrial TiOSO_4_ solution used in this work was obtained from a TiO_2_ pigment factory, and its primary compositions are TiO_2_ = 189 g/L, *m*(effective H_2_SO_4_)/*m*(TiO_2_) = 1.87. Commercial anatase TiO_2_ and rhodamine B (Rh.B) were obtained from Fuchen Chemical Reagent Co., Ltd. (Tianjin, China). Methylene blue (MB) was obtained from Chengdu Chron Chemical Reagent (Chengdu, China). Melamine was obtained from Tianjin Kemiou Chemical Reagent Co., Ltd. (Tianjin, China).

### 2.2 Preparation of S-TiO_2_ and Red TiO_2_

S-TiO_2_ was synthesized by using a hydrothermal route. In a typical synthesis, 92 mL of industrial TiOSO_4_ and 50 mL of water were heated to 96 ± 1 °C, respectively. Afterward, the TiOSO_4_ solution was dropped in preheated water within 20 min (using a peristaltic pump) under constant stirring. After mixing, the solution was poured into a 200 mL Teflon-lined autoclave and aged at 110 °C for 3 h. After the reaction, the precipitate was centrifuged, washed with deionized water, and dried at 60 °C. Finally, the solid was sintered in air at 400 °C for 2 h, with a heating rate of 10 °C/min. The resulting sample was labeled as ST.

**Figure 1:**
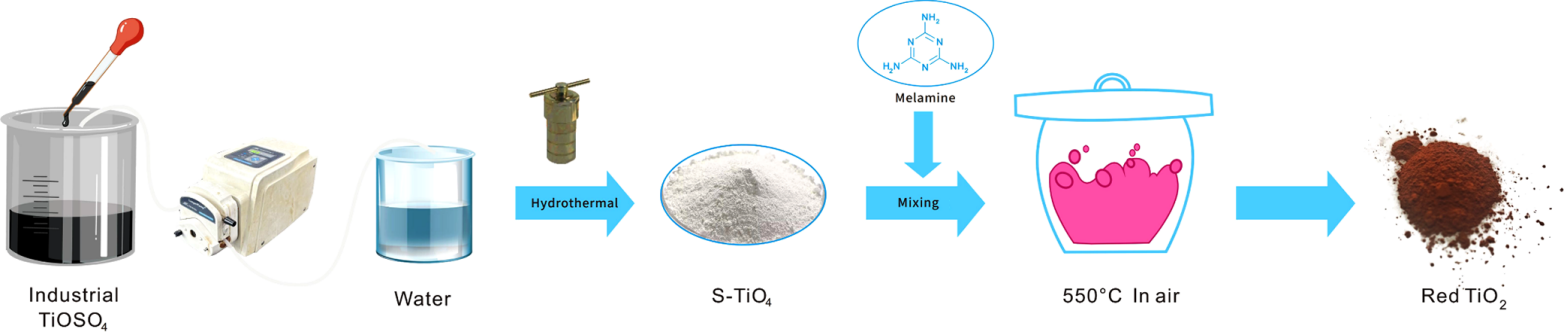
Schematic of the synthetic procedures of red TiO_2_.

The red TiO_2_ was prepared through simple high-temperature calcination in air. First, 0.5 g of the as-prepared ST and a certain amount (0.8, 1, 1.2, or 1.5 g) of melamine were ground for 20 min thoroughly. Subsequently, the mixture was placed in a 50 mL crucible, capped, and calcined at 550 °C for 2 h at a heating rate of 10 °C/min. The obtained products were labeled as SNCT-*X* (*X* was the mass of melamine). For comparison, 0.5 g of commercial anatase TiO_2_ and 1 g of melamine were ground and calcined under the same conditions (the product was denoted as CMT). The synthetic process of red TiO_2_ is depicted in Figure 1.

### 2.3 Photocatalytic Activity

The activity of ST and SNCT-*X* was evaluated by degrading 10 mg/L model compounds, Rh.B and MB. A 300 W xenon lamp with an optical filter to cut off the short-wavelength components was used as the light source (λ ≥ 420 nm). The horizontal distance between the catalyst and the lamp was 35 cm. For every photocatalytic experiment, 0.05 g of the catalyst was added to 100 mL of solution. Before irradiation, the mixed solution was kept in the dark for 30 min, and then, the lamp was turned on. A specified amount of the sample was taken and centrifuged at constant intervals. The supernatant solution was analyzed by using a Vis spectrophotometer (Model 721, Shanghai Xinmao Instrument Co., Ltd. China) at λ = 554 nm (for Rh.B) and 665 nm (for MB) to study the degradation extent of the model compound.

### 2.4 Electrochemical Performance Test

Transient photocurrent response and electrochemical impedance spectroscopy measurements were tested by an electrochemical workstation (CHI760E instruments, Shanghai Chenhua Instrument Co., Ltd. China) in a standard three-electrode setup with a Pt plate as the counter electrode, Ag/AgCl as the reference electrode, and FTO glass as the working electrode. The sample areas of 0.2826 cm^2^ of the working electrodes were immersed in the electrolyte. The electrolyte was 0.2 M Na_2_SO_3_. A 300 W Xe lamp as the light source (>420 nm) was used for the photocurrent tests.

### 2.5 Characterization

The X-ray powder diffraction (XRD) patterns were tested by the Malvern X’Pert3 Powder, with Cu Kα1 irradiation. Raman spectra were obtained by a Renishaw inVia with the wavenumber between 100 and 900 cm^–1^. Fourier transform infrared (FTIR) spectroscopy was tested by using a Thermo NICOLET 380. X-ray photoelectron spectroscopy (XPS) was carried out by a Thermo Scientific K-Alpha. Scanning electron microscopy (SEM) pictures were obtained on a ZEISS MERLIN SU8010 electron microscope. Transmission electron microscopy (TEM) images were taken using the FEI Tecnai F20 electron microscope. A Shimadzu UV-3600 spectrophotometer was used to test the optical properties. The surface area and pore size distributions of materials were measured using the Micromeritics ASAP 2460 nitrogen adsorption apparatus. The content of S was determined by a LECO CS230 carbon sulfur analyzer. The Bruker EMX PLUS electron paramagnetic resonance spectroscopy (EPR) was used to test the paramagnetic species.

### 2.6 First-Principles Simulation

First-principles simulation is selected to calculate formation energy and substitution formation by using CASTEP^32^ in the Materials Studio package. The ultrasoft pseudopotentials are selected to calculate the interaction between the electron and the nucleus. GGA and PBE functionals are used to describe the exchange-correlation energy of electrons. The lattice parameters were optimized and relaxed with a cutoff energy of about 314 eV. The *k*-point spacing of 0.07 Å^–1^ was used to sample the Brillouin zone. The energy is converged to 1.0 × 10^–6^ eV/atom.

## 3 Results and Discussion

### 3.1 Characterization of the Catalyst Structure and Properties

**Figure 2:**
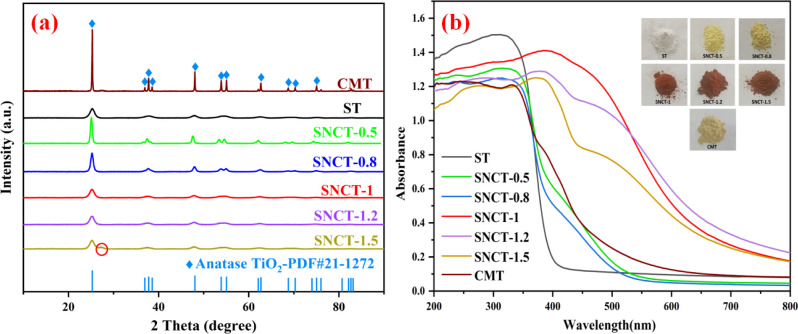
(a) XRD patterns of samples; (b) UV–vis spectra and optical photographs (inset) of different samples.

The XRD patterns are shown in Figure 2a. All the peaks of ST, CMT, and SNCT-*X* revealed similar characteristic peaks at 2 theta = 25.28°, 37.80°, 48.05°, 53.89°, 55.06°, and 62.68°, which correspond to anatase TiO_2_ (JCPDS card no. 21-1272).^33,34^ Moreover, the XRD patterns did not show any other impurities, except for the sample SNCT-1.5. Compared to the ST, the samples SNCT-0.5 and SNCT-0.8 have higher crystallinity, especially SNCT-0.5, in which the diffraction peak intensity increased and the width of the (101) crystal plane peaks became sharper. This is due to further high-temperature treatment of the sample ST, promoting an increase in its crystallinity. The peaks of SNCT-*X* become broader and weaker with the increase of melamine usage, indicating that the incorporation of nitrogen into TiO_2_ decreases its crystallinity. Previous studies have proved that doping with nitrogen will reduce the crystallinity of TiO_2_.^35,36^ This is probably due to the presence of defects on the grain boundary, which causes lattice strain and inhibits the grain growth.^37^ We can also see that there is a small peak at 2 theta = 27.4° for the sample SNCT-1.5. This is due to the excess melamine decomposition of the resulting g-C_3_N_4_.^38^ The grain sizes of samples were calculated using the Debye–Scherrer equation.^39^ The crystallite sizes of ST, SNCT-0.5, SNCT-0.8, SNCT-1, SNCT-1.2, and SNCT-1.5 are 6.8, 16.2, 12.3, 7.3, 7.2, and 6.8 nm, respectively.

UV–vis diffuse reflectance absorption of ST, SNCT-*X*, and CMT is shown in Figure 2b. In comparison to ST, all the SNCT-*X* samples showed a marked increase in absorption strength in the visible region. In addition, the visible absorption intensity changes significantly with the increase in melamine dosage. The samples SNCT-0.5 and SNCT-0.8 only exhibited tail shoulder absorption, which is similar to previous reports of nitrogen-doped titanium dioxide.^40,41^ The sample CMT also shows tail shoulder absorption in the visible region, similar to that of SNCT-0.5 and SNCT-0.8. With the further increase in the mass of melamine, the samples showed the overall translational absorption, which is probably because of the formation of band-to-band absorption.^42,43^ The samples SNCT-1 exhibited the best visible light absorption, much better than ST and CMT. Further increasing the amount of melamine (the samples SNCT-1.2 and SNCT-1.5) results in a decrease in light absorption, possibly due to excess melamine decomposition leading to residual organic matter in the sample. The optical photographs of ST, SNCT-*X*, and CMT are also shown in Figure 2b. The CMT shows a pale yellow color, similar in color to the well-known nitrogen-doped TiO_2_. For the sample SNCT-*X*, with the increase of melamine content, the color changed from the white of ST to pale yellow and further to red. We believe that the red color of the samples is attributed to the synergetic effects of Ti^3+^, oxygen vacancy, and high nitrogen doping, which will be discussed later.

**Figure 3:**
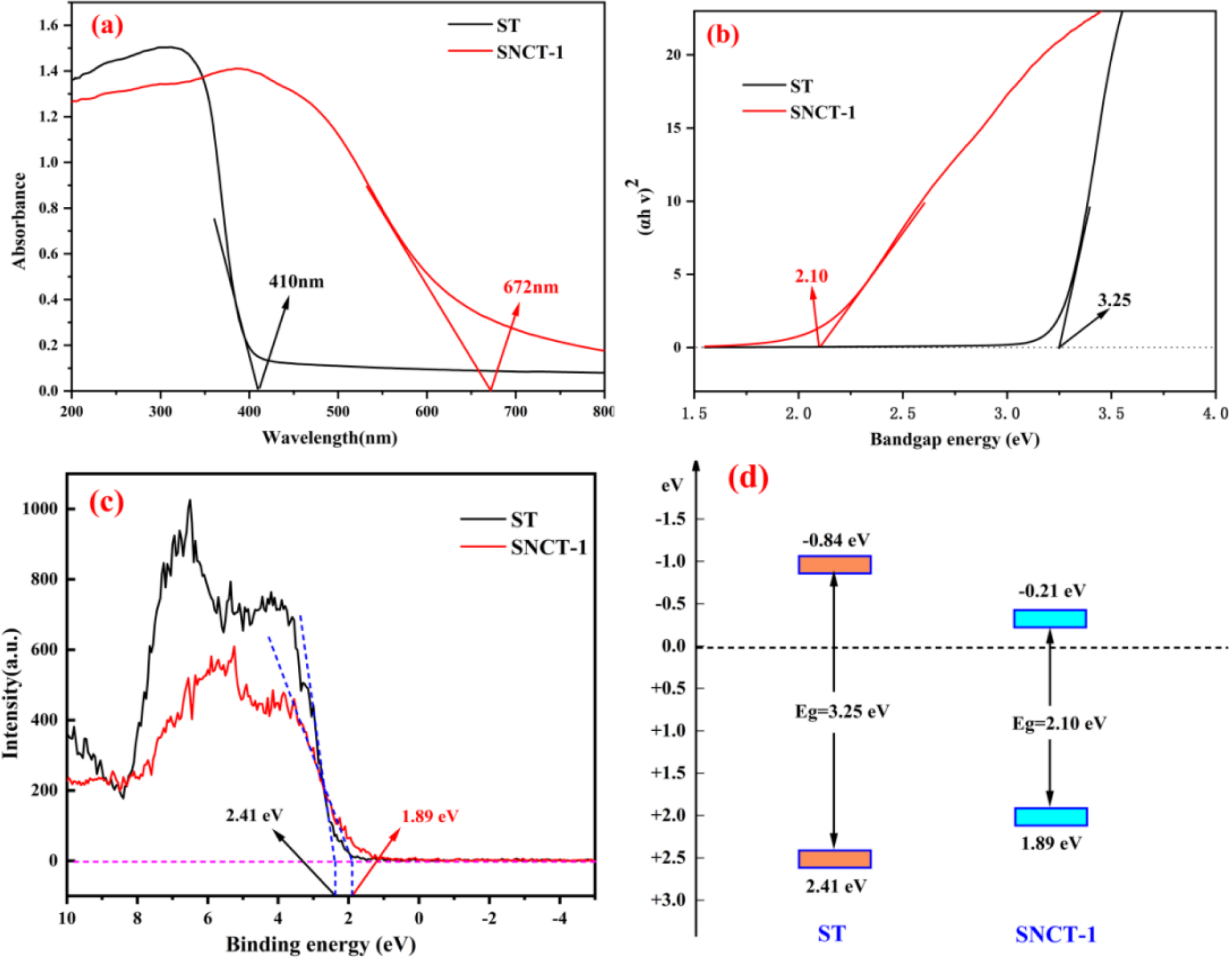
(a) UV–vis spectra of ST and SNCT-1; (b) bandgap energies of ST and SNCT-1; (c) XPS valence band spectrum of ST and SNCT-1; (d) band structures of ST and SNCT-1.

In order to more intuitively compare the optical properties of white and red TiO_2_, Figure 3 shows the light absorption and bandgap properties of ST and SNCT-1. As depicted in Figure 3a, compared to the ST with an absorption edge of about 410 nm, the absorption edge of SNCT-1 is extended to about 672 nm, which almost covers the total visible region. The bandgap of samples was estimated using the (α*h*ν)^2^–*h*ν relationship^44^ and is shown in Figure 3b. In comparison to ST (bandgap energy of 3.25 eV), SNCT-1 has a much lower optical bandgap (2.10 eV), which is beneficial for visible light responsiveness. Figure 3c shows the XPS valence band spectra of ST and SNCT-1, and the obtained valence band (VB) values are 2.41 and 1.89 eV, respectively. Because the bandgap of the sample is bound, the bottom potential of the conduction band (CB) of ST and SNCT-1 can be deduced to be −0.84 eV and −0.21 eV, respectively. The corresponding band structures are illustrated in Figure 3d. Compared to ST, the VB position of SNCT-1 moves in the direction of the negative potential by 0.52 eV, and the CB position shifts in the direction of the positive potential by 0.63 eV.

**Figure 4:**
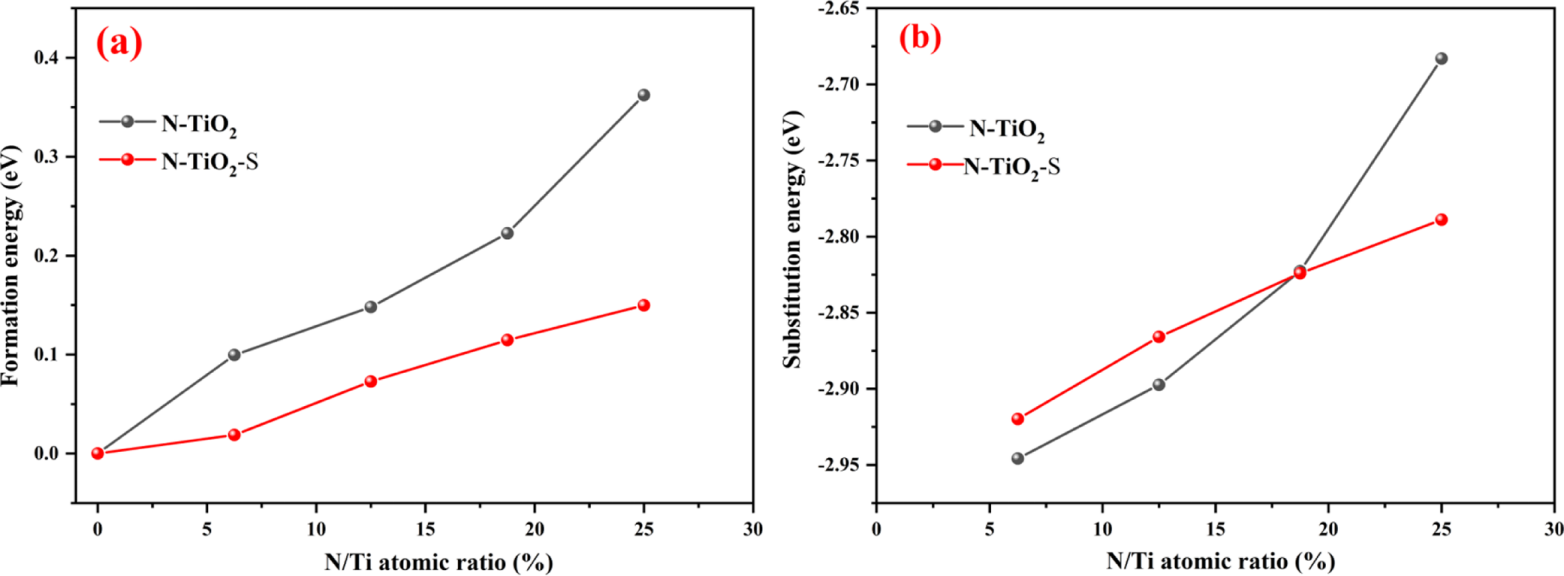
(a) Formation energy and (b) substitution energy of nitrogen-doped TiO_2_ with and without S.

First-principles simulation results are shown in Figure 4. As depicted in Figure 4a, the calculated formation energy of nitrogen-doped TiO_2_ at different N/Ti ratios is obviously reduced by the existence of S. In particular, the formation energy significantly decreases from 0.36 to 0.15 eV at the N/Ti ratio of 25 at. %, even falling by more than half. The calculated substitution energy (Figure 4b) of nitrogen-doped TiO_2_ shows a different trend. At low N/Ti ratios (6.25 and 12.5 at. %), the substitution energy of nitrogen-doped TiO_2_ with S is higher than that without S, but at high N/Ti ratios (18.75 and 25 at. %), the opposite is true. These results indicate that predoped S is beneficial for the preparation of highly nitrogen-doped TiO_2_.

**Figure 5:**
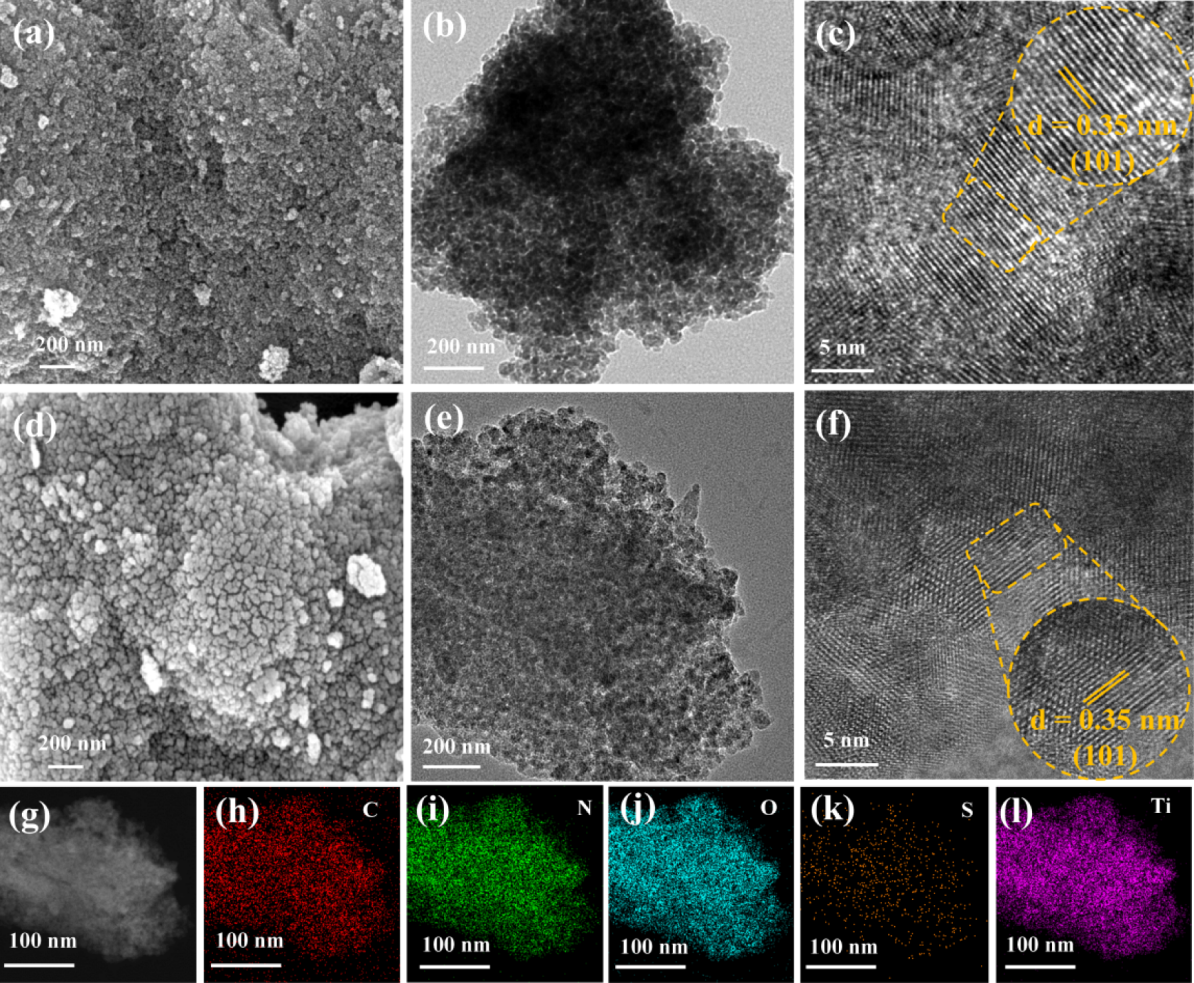
SEM, TEM, and HRTEM images of ST (a–c) and SNCT-1 (d–f); STEM image of SNCT-1 and the corresponding EDS mappings of C, N, O, S, and Ti (g–l).

The morphology of the samples was analyzed by SEM. Figure 5a,d reveals the SEM images of ST and SNCT-1. It is seen that the two samples exhibited a spherical shape and a high extent of agglomeration. Further morphology analysis was conducted using TEM and HRTEM. As depicted in Figure 5b,e, the samples ST and SNCT-1 have similar TEM images. The diameters of ST and SNCT-1 were ∼7 nm, which is similar to XRD analysis. The HRTEM images (Figure 5c,f) clearly show the crystal lattice of ST and SNCT-1. The ∼0.35 nm interplanar spacing corresponds to the (101) plane of anatase TiO_2_. Figure 5g–l illustrates the energy dispersive spectra (EDS) of SNCT-1. Apparently, there is a copresence of Ti, O, N, and S elements in SNCT-1, but the distribution of sulfur is relatively sparse, which is due to the high-temperature calcination resulting in the escape of sulfur. In addition, the distribution of the N element in the sample is relatively uniform, which proves that TiO_2_ is doped in the sample.

**Figure 6:**
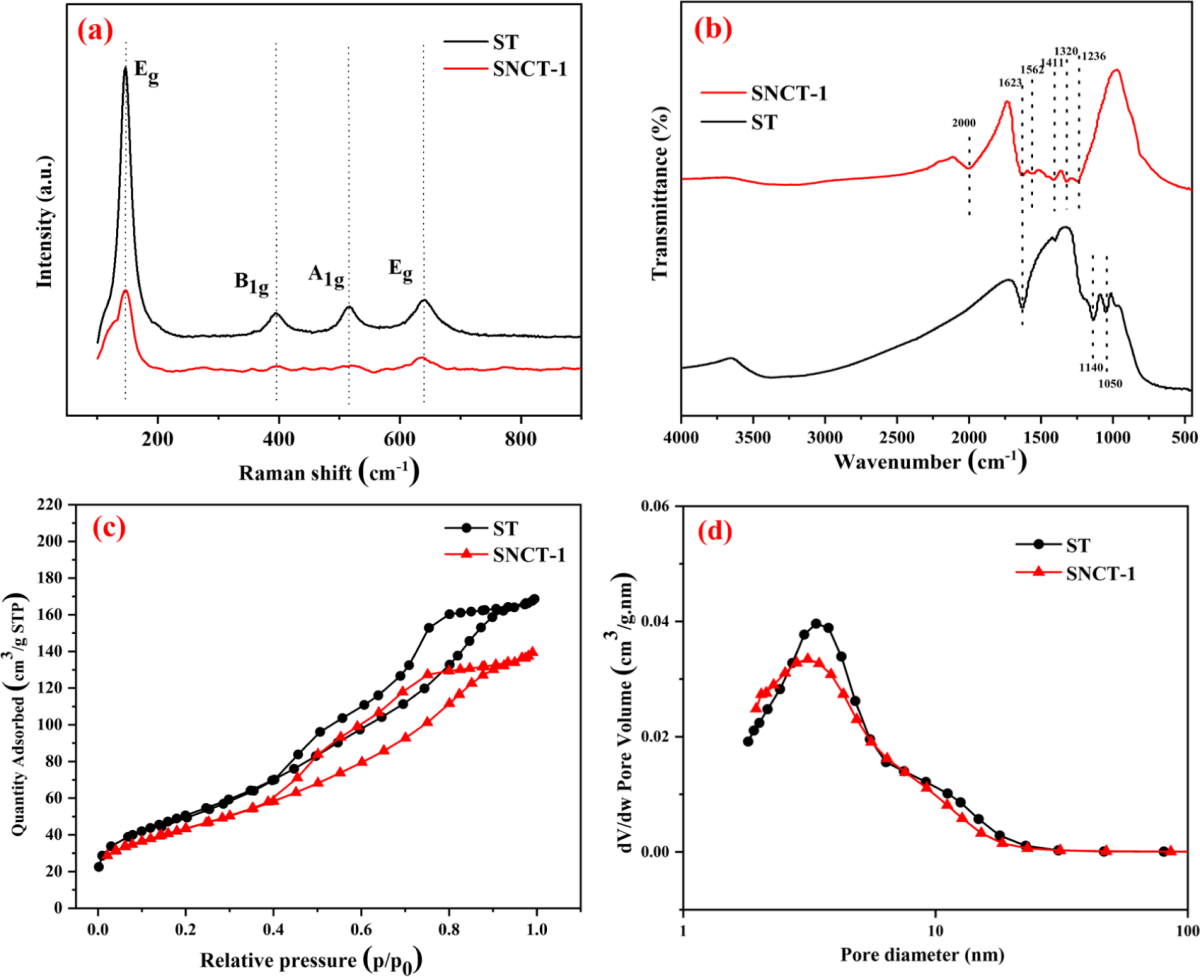
A comparison of Raman spectra (a), FTIR spectra (b), nitrogen isotherms (c), and pore size distribution (d) of ST and SNCT-1.

Raman spectra were used to explore the crystal properties of white and red TiO_2_. Figure 6a shows the Raman spectra of ST and SNCT-1. The two samples have similar Raman spectra, and both have four apparent vibrational peaks at 147 cm^–1^, 395 cm^–1^, 515 cm^–1^, and 640 cm^–1^. These peaks correspond to anatase TiO_2_,^45^ indicating that ST and SNCT-1 consist of a pure anatase phase. In addition, the peak strength of SNCT-1 is significantly lower than that of ST, which may be due to nitrogen doping leading to the formation of defects on the grain boundary of TiO_2._ It has been shown that the material defects can affect the vibration mode of Raman.^46^

The FT-IR spectra of ST and SNCT-1 are revealed in Figure 6b. The absorption peak at 3424 cm^–1^ belongs to the stretching vibrations of the hydroxyl group, and the peak at 1623 cm^–1^ represents the bending vibrations of surface-adsorbed water.^47^ The peak at about 1140 cm^–1^ belongs to the stretching vibrations of the S–O bond, and the band at 1050 cm^–1^ represents the Ti–O–S peak, which confirms that S exists in the ST sample.^48^ These two peaks disappear completely in the SNCT-1 sample, which is because the high-temperature calcination of ST leads to the escape of S.^49^ A broad peak between 700 and 1000 cm^–1^ is the librational band of adsorbed water.^50^ The absorption band between 400 and 800 cm^–1^ belongs to Ti–O stretching vibration.^51^ Compared to ST, SNCT-1 shows new peaks at 1236–1562 cm^–1^, mainly belonging to the stretching and bending vibrations of N–H and C–N,^52,53^ which confirm that the N element is successfully doped into TiO_2_.

The surface areas and pore size distributions were tested by N_2_ adsorption–desorption measurements. As depicted in Figure 6c, the typical class IV Langmuir adsorption–desorption isotherm with H3 hysteresis loops exists for ST and SNCT-1, which indicates that mesopores are formed in both samples.^54^ The surface areas of ST and SNCT-1 are approximately 186.8 m^2^/g and 157.7 m^2^/g. The decrease of surface areas for SNCT-1 is due to the high-temperature calcination. Figure 6d shows the pore size distributions of ST and SNCT-1. Obviously, ST and SNCT-1 have similar pore size distributions, and their average pore diameters (calculated by the BJH method) are 4.2 and 5.2 nm. The BJH adsorption cumulative pore volumes of ST and SNCT-1 were 0.258151 and 0.225901 cm^3^/g, respectively.

**Figure 7:**
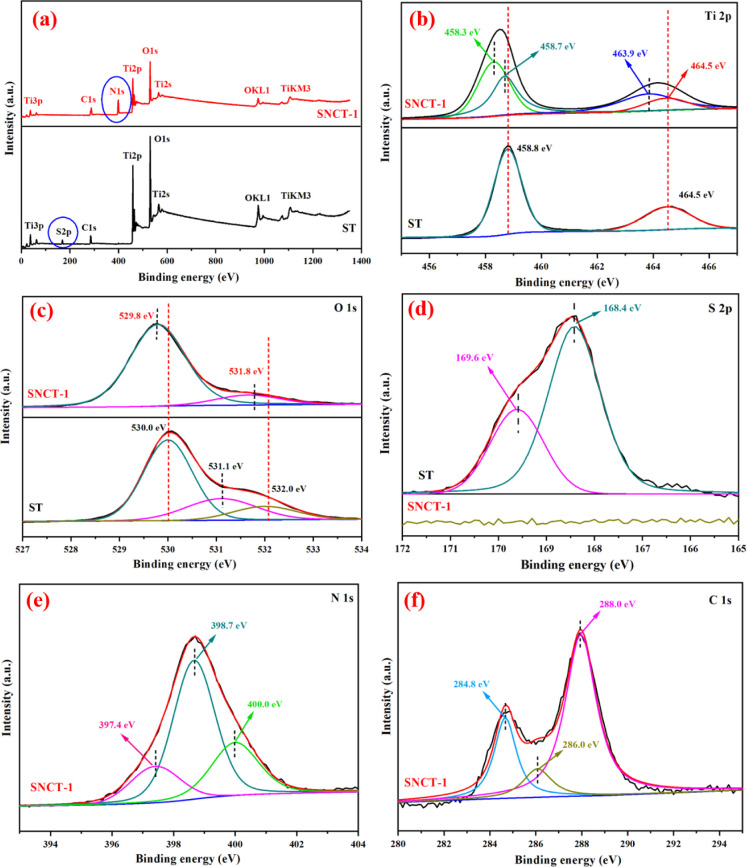
XPS spectra of ST and SNCT-1 (a); high-resolution XPS spectra of Ti 2p (b), O 1s (c), S 2p (d), N 1s (e), and C 1s (f).

The surface elements and oxidation states were analyzed using XPS. As displayed in Figure 7a, the survey spectrum of ST clearly showed the peaks of Ti, O, S, and C; however, the survey spectrum of SNCT-1 clearly showed the peaks of Ti, O, N, and C. This result shows that the N element was successfully doped into titanium dioxide. The S peak did not exist in SNCT-1 due to its low content, which is consistent with the EDS analysis and FT-IR analysis.

The Ti 2p spectra of ST and SNCT-1 are shown in Figure 7b. The Ti 2p peak of ST is only divided into two peaks at 464.5 and 458.8 eV, which belong to Ti^4+^ 2p_1/2_ and Ti^4+^ 2p_3/2_, respectively.^55^ For SNCT-1, the Ti 2p peaks shift toward lower binding energy and can be divided into two more peaks at 458.3 and 463.9 eV, which can be assigned to Ti^3+^ 2p_3/2_ and Ti^3+^ 2p_1/2_.^56^

From Figure 7c, the O 1s spectrum of ST was divided into three peaks at 530.0 eV (belonging to lattice oxygen such as the Ti–O bond), 531.1 eV (belonging to oxygen in SO_4_^2–^),^57^ and 532.0 eV (assigned to surface hydroxyl groups or absorbed water).^58^ The O 1s spectrum of SNCT-1 was divided into two peaks at 529.8 and 531.8 eV, which correspond to the peaks of 530.0 and 532.0 eV of the ST sample, but shifted by about 0.2 eV, may be due to the doping of the N element. The disappearance of the O 1s peak at about 531.1 eV in SNCT-1 is because of the escape of sulfur.

Figure 7d shows the S 2p XPS spectra of ST and SNCT-1. The S 2p XPS spectra of SNCT-1 have no obvious signal due to the high-temperature calcination of ST, which leads to the escape of S. The S 2p XPS spectra of ST could be fitted with two peaks at 168.4 and 169.6 eV, which correspond to S 2p_3/2_ and S 2p_1/2_, respectively. Generally, these peaks are ascribed to S^6+^ such as sulfur in SO_4_^2–^.^59,60^ The N 1s XPS spectra of the sample SNCT-1 could be fitted with three peaks (shown in Figure 7e). The peak at 397.4 eV can be assigned to the Ti–N bond.^61^ The peak at 398.7 eV is commonly referred to as oxygen atoms being replaced by N atoms to form the N–Ti–N bond.^40^ In addition, the peak at 400.0 eV is referred to as the Ti–O–N bond.^62^ The high-resolution C 1s spectra of the samples SNCT-1 were divided into three different peaks (Figure 7f). The peak at 284.8 eV can be attributed to the adventitious carbon pollution in the XPS measurement, whereas the peaks at 286.0 and 288.0 eV correspond to the C–O bond and C=O bond, respectively.^63^

### 3.2 Photocatalytic Reaction

**Figure 8:**
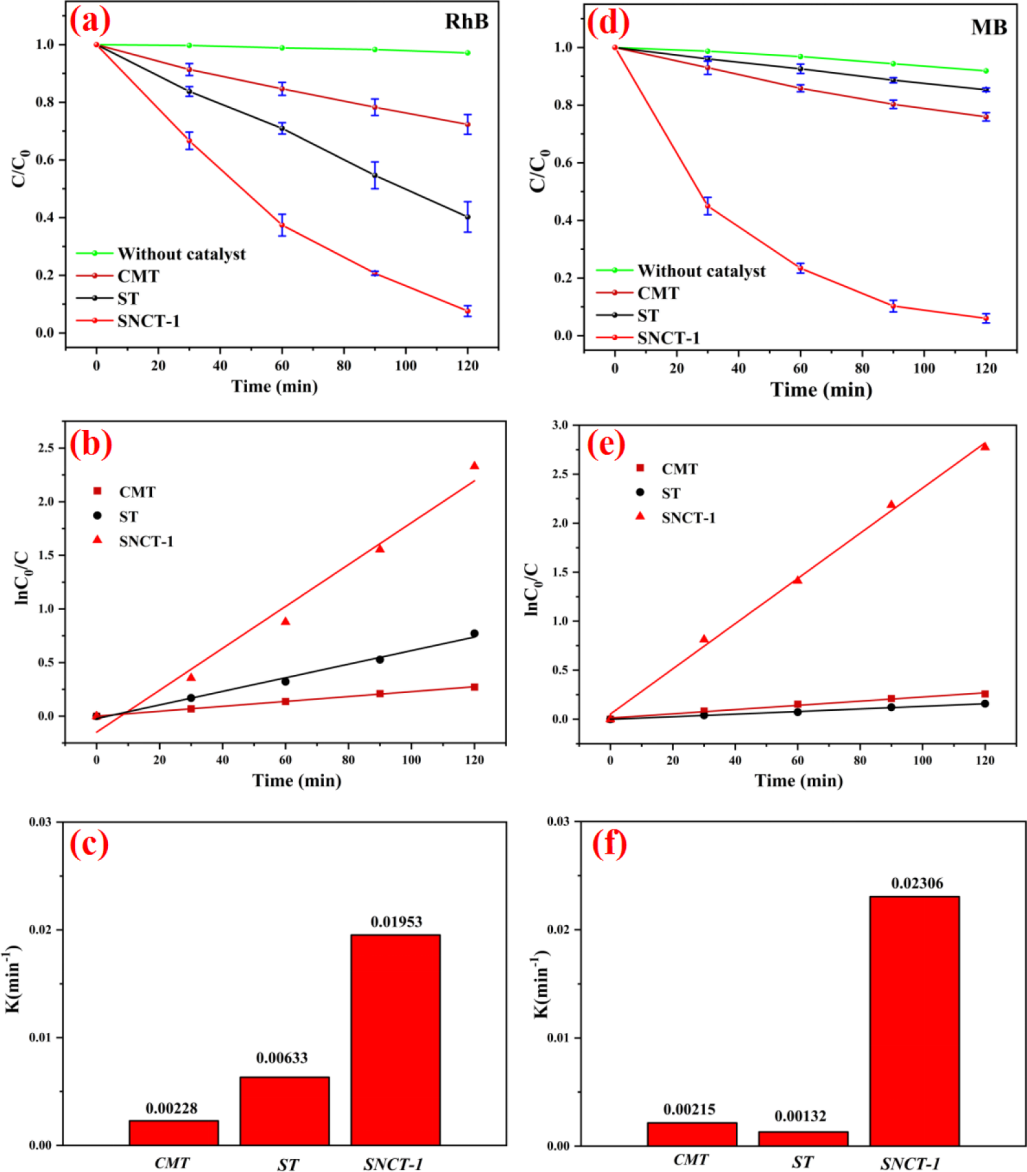
(a) Comparison of photocatalytic activity for Rh.B under visible light and corresponding kinetic curves (b) and degradation rate constants (c); (d) comparison of photocatalytic activity for MB under visible light and corresponding kinetic curves (e) and degradation rate constants (f).

The photocatalytic activity of ST and SNCT-1 was evaluated toward the decolorization of Rh.B and MB. For comparison, the activity of CMT was also evaluated under the same conditions. Figure 8a reveals the photocatalytic degradation activity of Rh.B. Apparently, only negligible degradation of Rh.B was observed without a catalyst, indicating that Rh.B is relatively stable. Compared to CMT and ST, SNCT-1 shows better photocatalytic activity in the visible light degradation of Rh.B. As can be seen from Figure 8b, the linear dynamics curve of ln(*C*_0_/*C*) vs time is consistent with the first-order dynamics of the Langmuir–Hinshelwood (L–H) model, indicating that photodegradation is a pseudo-first-order reaction.^64^ The calculated values of rate constants for all of the samples are shown in Figure 8c. Obviously, the SNCT-1 sample exhibited the highest rate constant (0.01953 min^–1^), which is far above that of the ST (0.00633 min^–1^) and CMT (0.00228 min^–1^).

The photocatalytic degradation of MB is provided in Figure 8d. Compared to CMT and ST, SNCT-1 also shows obviously better photocatalytic activity in the visible light degradation of MB. As can be seen from Figure 8e, the linear dynamics curve of ln(*C*_0_/*C*) vs time is consistent with the first-order dynamics of the Langmuir–Hinshelwood (L–H) model. The calculated values of rate constants for all of the samples are shown in Figure 8f. The SNCT-1 sample exhibited the highest rate constant (0.02306 min^–1^), which is far above those of the ST (0.00132 min^–1^) and CMT (0.00215 min^–1^).

To sum up, SNCT-1 shows much better photocatalytic activity in the visible light degradation of Rh.B and MB, especially in the degradation of MB. The excellent photocatalytic activity of the SNCT-1 sample is attributed to the lower bandgap, which greatly improves the visible light absorption.

**Figure 9:**
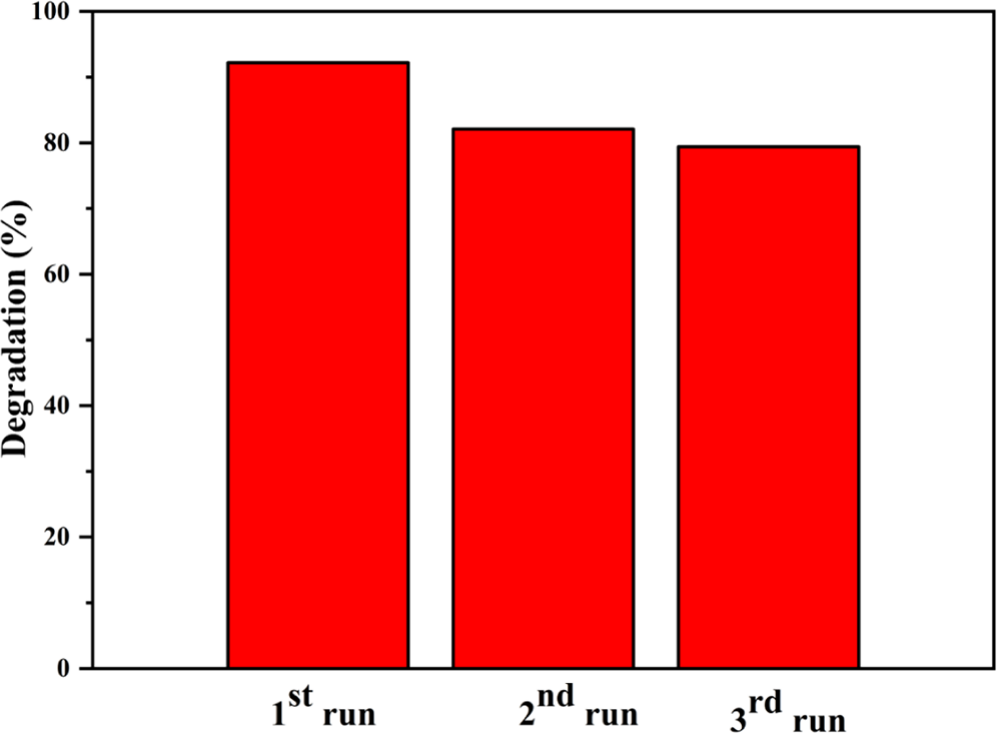
Cycling stability of SNCT-1 for the photocatalytic degradation of Rh.B.

The stability of the SNCT-1 sample was investigated by cycle experiments, and the results are shown in Figure 9. The sample was recycled from the solution by centrifugation, washed, and dried at 80 °C for 10 h between tests during the cycling stability experiments. The photodegradation efficiencies of Rh.B were 92.2%, 82.1%, and 79.4% in the three cycles, respectively. The decrease in sample activity may be caused by partial inactivation of the catalyst.

### 3.3 Photocatalytic Degradation Mechanism

**Figure 10:**
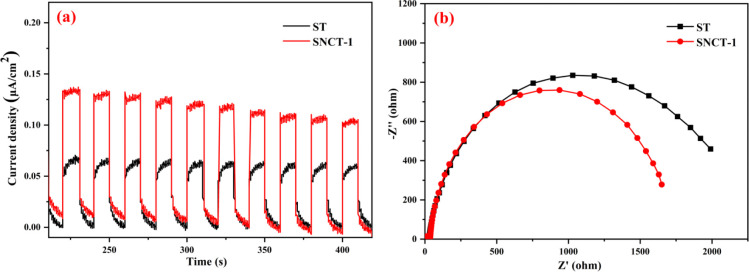
(a) Transient photocurrent response of ST and SNCT-1; (b) EIS spectra of ST and SNCT-1.

Electrochemical performance was measured to better understand the reason for the increased photocatalytic behaviors. The transient photocurrent response curves are shown in Figure 10a. We can clearly see that SNCT-1 produced a higher photocurrent than ST, suggesting that SNCT-1 could more efficiently harvest solar light. In general, higher photocurrent values will lead to higher photocatalytic activity.^65^Figure 10b illustrates the electrochemical impedance spectroscopy (EIS) profiles of ST and SNCT-1. Compared with ST, SNCT-1 exhibited a smaller radius of EIS, suggesting that the N-doping reduced the internal carrier transfer resistance and further speeded up electron transport.

**Figure 11:**
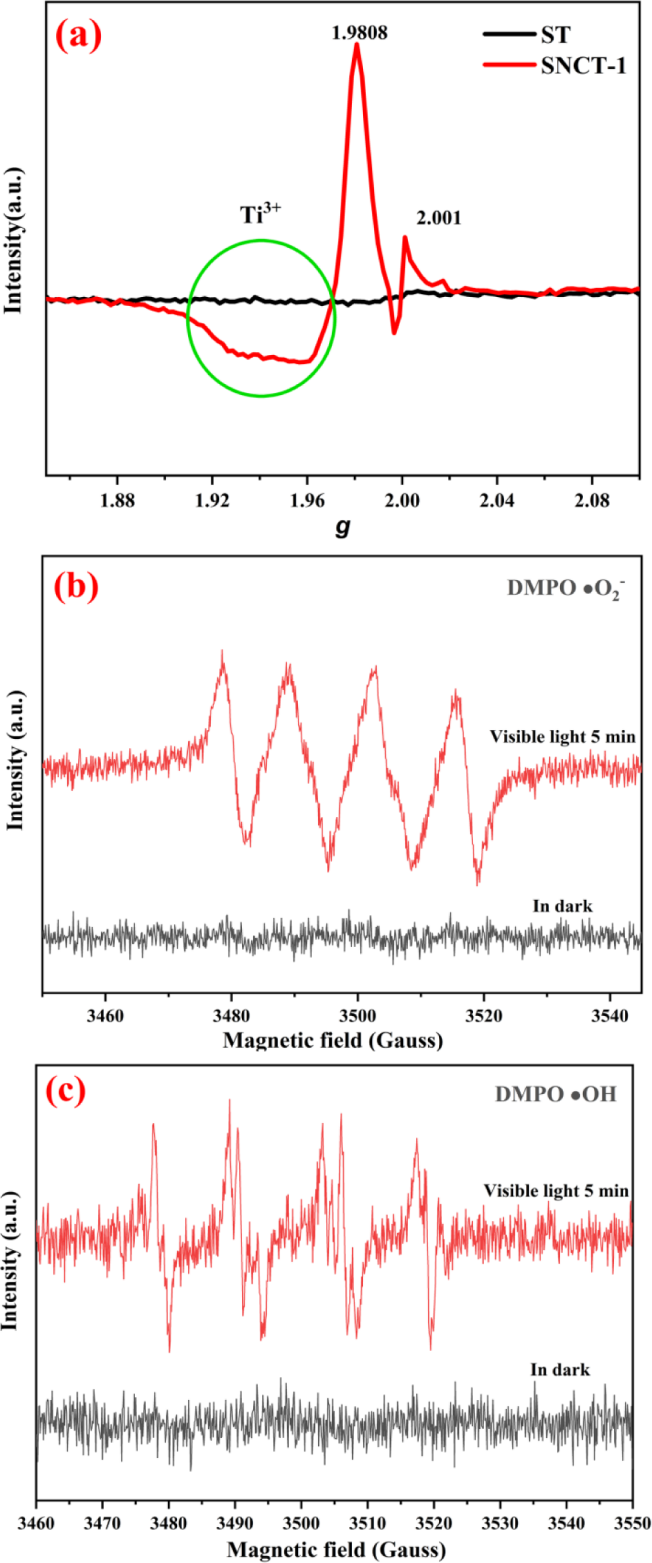
(a) EPR spectra of ST and SNCT-1; (b) EPR spectra of DMPO-•O^2–^ in the methanol dispersion of SNCT-1; (c) EPR spectra of DMPO-•OH in aqueous dispersions of SNCT-1.

Room-temperature EPR was used to assess the oxygen vacancies in ST and SNCT-1. As depicted in Figure 11a, there is almost no EPR signal measured in ST, whereas there is a significant signal in the SNCT-1 sample. Apparently, SNCT-1 has two obvious EPR peaks at *g* = 2.0010 and *g* = 1.9813. The former is widely recognized to be ascribed to oxygen vacancies.^66,67^ The latter can be noted because of Ti^3+^.^14,68^ These results demonstrated that treating ST with melamine resulted in the formation of a large number of oxygen vacancies and Ti^3+^. Research shows that oxygen vacancies and Ti^3+^ can produce impurity levels just below the CB, which leads to increased visible light absorption.^69^ In addition, we think that the red color of SNCT-1 mostly originates from the Ti^3+^ and oxygen vacancies.

The experiment of DMPO-EPR measurements is further used to detect the generation of active species under illumination (Figure 11(b,c). As shown in Figure 11b, no characteristic signals could be detected in the dark, indicating that no active species are generated in the dark, while there are obviously •O^2–^ signals under visible light for 5 min, indicating that •O^2–^ are formed.^70^Figure 11c shows no obvious •OH signals in the dark, but strong •OH signals are detected under visible light irradiation.^71^ These experiments confirm that •OH and •O^2–^ radicals are the important active species for the photocatalysis of SNCT-1.

**Figure 12:**
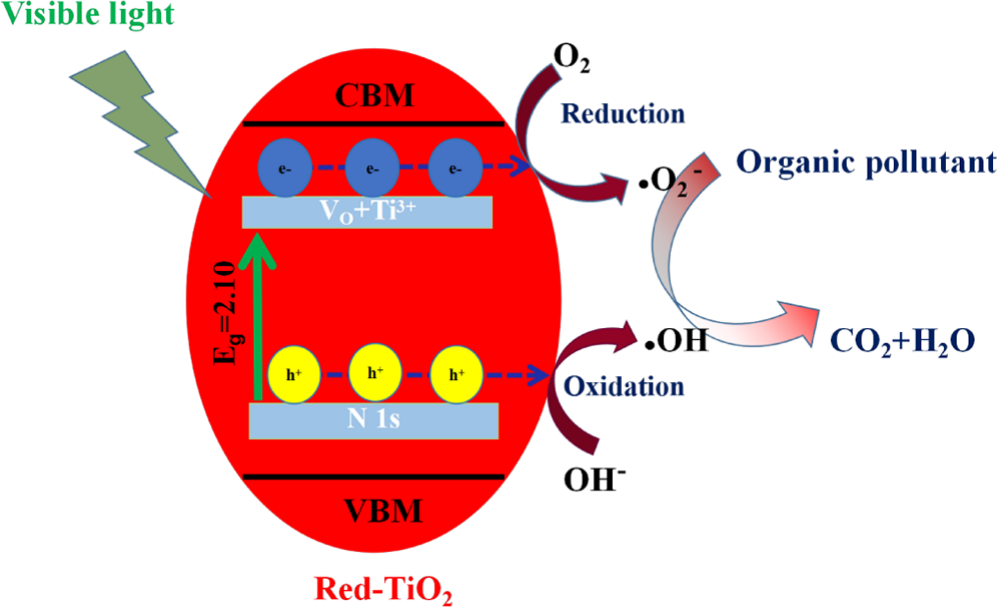
Mechanism of red-TiO_2_ bandgap narrowing and the photocatalytic mechanism.

The photocatalytic mechanism of red-TiO_2_ was put forward and is shown in Figure 12. EDS-mapping and XPS analysis have confirmed that the red-TiO_2_ contains large amounts of nitrogen. A new energy level of the N 2p band can be formed above the VB of the O 2p, which reduces the bandgap of TiO_2_. Furthermore, according to the results of EPR and XPS, the red-TiO_2_ produces abundant oxygen vacancies and Ti^3+^, which lead to the formation of an intermediate energy level below the conduction band.^72^ The coexistence of N 1s energy levels, oxygen vacanies, and Ti^3+^ makes the red-TiO_2_ have excellent visible light absorption, thus efficiently separating the photogenerated electron–hole pairs. Under visible light irradiation, the excited electrons and adsorbed oxygen can form superoxide anion radicals (•O^2–^).^73^ In the meantime, the holes can oxidize surface hydroxide to generate hydroxyl radicals (•OH).^74^ Therefore, Rh.B and MB can be degraded by these active species.

## 4 Conclusions

We have used a novel route to synthesize a red-TiO_2_ photocatalyst by first synthesizing S-doped TiO_2_ and then calcinating it with melamine. The predoped S could decrease the formation energy and substitution formation of nitrogen-doped TiO_2_, which is beneficial for high nitrogen doping. The red-TiO_2_ has a small particle size (around 7 nm) and a low bandgap (2.10 eV), which exhibits excellent visible light absorption. EPR and XPS analyses show the red-TiO_2_ with abundant oxygen vacancies and Ti^3+^. The synergetic effect of Ti^3+^, oxygen vacancies, and nonmetallic elements leads to the bandgap narrowing of TiO_2_. The red-TiO_2_ exhibits much better photocatalytic activity in the visible light degradation of Rh.B and MB.
